# Clinical characteristics of HIV-1-infected patients with high levels of plasma interferon-γ: a multicenter observational study

**DOI:** 10.1186/s12879-018-3643-2

**Published:** 2019-01-05

**Authors:** Dai Watanabe, Tomoko Uehira, Sachiko Suzuki, Erina Matsumoto, Takashi Ueji, Kazuyuki Hirota, Rumi Minami, Soichiro Takahama, Kimikazu Hayashi, Morio Sawamura, Masahiro Yamamoto, Takuma Shirasaka

**Affiliations:** 1 AIDS Medical Center, National Hospital Organization Osaka National Hospital, 2-1-14, Hoenzaka, Chuo-ku, Osaka City, Osaka 540-0006 Japan; 2 Department of Advanced Medicine for HIV Infection, Osaka University Graduate School of Medicine, 2-2, Yamada-oka, Suita City, Osaka 565-0871 Japan; 3 Internal Medicine, Clinical Research Institute, National Hospital Organization, Kyushu Medical Center, 1-8-1, Jigyohama, Chuo-ku Fukuoka City, Fukuoka 810-8563 Japan; 4 Department of Obstetrics and Gynecology, National Organization Kanmon Medical Center, 1-1, Chofusotouracho, Shimonoseki City, Yamaguchi 752-8510 Japan; 5 Department of Clinical Research, National Hospital Organization Shibukawa Medical Center, 383, Shiroi, Shibukawa City, Gunma 377-0280 Japan

**Keywords:** HIV-1 infection, Interferon-γ, Interleukin-6, CD4^+^ cell count recovery

## Abstract

**Background:**

Circulating interferon-γ (IFN-γ) concentration may be sustained at a high level regardless of the initiation of antiretroviral therapy (ART) in some patients with HIV-1 infection. In the present study, we examined the clinical characteristics of HIV-1-infected patients with high levels of plasma IFN-γ.

**Methods:**

The study subjects were patients infected with HIV-1 who were either naïve to ART with CD4^+^ cell count > 200 cells/μL (*n* = 12), or had achieved viral suppression after ART for over a year (*n* = 188). The levels of plasma IFN-γ and interleukin-6 (IL-6) were measured by the enzyme-linked immunosorbent assay. Patients were divided into high IFN-γ and low IFN-γ groups based on a cutoff level of 5 pg/mL.

**Results:**

The high IFN-γ group included 41 patients (21%). Compared to the patients on ART with low IFN-γ levels, those on ART in the high IFN-γ group were more likely to be younger than 50 years of age (*P* = 0.0051) and less likely to have dyslipidemia (*P* = 0.0476) or to be on a protease inhibitor (*P* = 0.0449). There was no significant difference between groups in the median increase of CD4^+^ cell counts from the initiation of ART for up to 3 years. However, after 4 years, the increase in CD4^+^ cell counts was significantly lower in the high IFN-γ group compared with that in the low IFN-γ group. There were no such significant differences between patients with low and high (> 2 pg/mL) levels of plasma IL-6.

**Conclusion:**

We concluded that HIV-1-infected patients with high levels of circulating IFN-γ did not have a higher rate of comorbidities related to immune activation. However, they exhibited lower CD4^+^ cell count recovery after 4 years of being on ART. This deficit could be a consequence of persistent immune activation.

**Electronic supplementary material:**

The online version of this article (10.1186/s12879-018-3643-2) contains supplementary material, which is available to authorized users.

## Background

During HIV-1 infection, serum cytokine levels generally rise with the progression of immunodeficiency and decrease with the initiation of antiretroviral therapy (ART) [1–6]. However, we previously measured the levels of 12 serum cytokines in 35 HIV-infected patients and demonstrated that the level of serum interferon-γ (IFN-γ) exhibits a trend different from those of other cytokines [7]. Specifically, in a cross-sectional study, we revealed that some patients who were asymptomatic carriers or being treated by ART had a high level of serum IFN-γ. Similarly, in a longitudinal study, we revealed that a high level of serum IFN-γ was sustained in about 30% of the patients after the initiation of ART. Those observations suggested that serum IFN-γ concentration is maintained at a high level in some patients regardless of the state of immunodeficiency or ART. Thus, compared with the concentrations of other cytokines, the level of circulating IFN-γ may have different clinical significance in patients infected with HIV-1 [8].

It has been reported that initiation of ART reduces the levels of inflammation-associated soluble biomarkers, including serum cytokines such as interleukin-6 (IL-6), although their levels do not return to those observed in non-infected population [3, 5, 9]. Various factors, such as old age, comorbidities, and death, have been associated with the high level of circulating IL-6 in patients infected with HIV-1 [9–11].

In contrast to the current knowledge about factors influencing IL-6 levels, the parameters associated with the high level of circulating IFN-γ have not been well established. It is unclear whether sustained high level of circulating IFN-γ has any influence on the clinical course of individuals infected with HIV-1. Although it may have an immunostimulatory effect that suppresses viral replication and increases CD4^+^ cell count, excessive immune activation may result in the reduction of CD4^+^ cell count and increased likelihood of comorbidity development. In the present study, we examined HIV-1-infected patients who were either naïve to ART, with CD4^+^ T cell count > 200 cells/μL, or had HIV-1 RNA levels below the detection limit after being on ART for over a year and compared the clinical characteristics between the participants with high and low levels of plasma IFN-γ or IL-6.

## Methods

### Study population

The study complied with the principles of the Declaration of Helsinki regarding the investigations in human subjects and was performed after an ethics approval H26-NHO (AIDS)-03 had been received from Central Institutional Review Board Committee of the National Hospital Organization of Japan. Written consent was obtained from all study participants. The study cohort comprised 200 HIV-1-infected patients over 20 years of age who were regularly seen at one of the four hospitals of the National Hospital Organization. Patients were either naïve to ART, with CD4^+^ T cell count > 200 cells/μL, or had achieved viral suppression after having been on ART for over a year. Patients who developed fever or had other acute diseases were excluded from the study cohort.

### Plasma measurements of IFN-γ and IL-6

Plasma was separated from the whole blood and kept frozen in − 80 °C until use. The levels of plasma IFN-γ and IL-6 were measured by the enzyme-linked immunosorbent assay (ELISA) (ThermoFisher Scientific, Waltham, MA) according to the supplier’s protocol. A cutoff level of 5 pg/mL was selected to delineate groups with high and low IFN-γ levels as previously described [7]. The cutoff level for IL-6 was set to 2 pg/mL, close to the upper quartile (1.9 pg/mL). When repeated blood collections were possible for patients with IFN-γ ≥5 pg/mL, samples were collected three times with at least 1 month in between each collection to confirm the reproducibility of high plasma IFN-γ levels.

### Comparisons of the groups with high and low levels of IFN-γ and IL-6

Participants were divided into the groups with high and low IFN-γ and IL-6 levels based on cutoff values indicated above. The initial measurement of IFN-γ was used to divide the patients into the groups. Sex, age, assumed route of infection, use of ART, and comorbidities were compared at the time of entry to the study. The patients were defined to have diabetes mellitus, dyslipidemia, hypertension, chronic kidney disease, and osteoporosis if they were diagnosed with these conditions according to the Japan Diabetes Society criteria, Japan Atherosclerosis Society guideline, Japanese Society of Hypertension guideline, Japanese Society of Nephrology guideline, and Japanese Society for Bone and Mineral Research criteria [12–14], respectively. Patients that received treatment for these conditions were also included. History of malignancy was defined as having history of any cancer that had been proven by biopsy. Chronic hepatitis B was defined as persistence of hepatitis B surface antigen for 6 months or more. Chronic hepatitis C was defined as the presence of detectable hepatitis C virus RNA in the serum. Only those comorbidities that were identified in over 5% of the study patients at entry were considered for analysis. Clinical categories, number of CD4^+^ T cells (CD4^+^ cell count), plasma HIV-1-RNA levels, and comorbidities at the time of HIV-1 diagnosis were also collected retrospectively from the medical records and compared between the groups. The change in CD4^+^ cell count following ART was calculated based on the cell count prior to the initiation of ART. Changes in CD4^+^ cell count were measured from 1 year after ART initiation for up to 10 years.

### Phylogenetic analysis

The results of the drug resistance test for HIV-1 were collected retrospectively for the purpose of the study. Resistance testing was performed as described previously [15]. Briefly, viral RNA was extracted from a plasma sample. The regions of HIV-1 protease and reverse transcriptase were amplified by using the reverse transcription polymerase chain reaction (PCR) method followed by nested PCR. After the purification of the amplified PCR products, the sequences were obtained by direct sequencing. Sequences used in the study (protease amino acid residues 1–99 and reverse transcriptase amino acid residues 1–240) were registered in GenBank (Additional file 1: Supplementary Materials and Methods). Nucleic acid sequences were aligned using Clustal W program version 2.1 (http://www.clustal.org/clustal2/) and GENETYX-MAC version 18.0.6 (GENETYX, Tokyo, Japan). HIV-1 subtype B (HXB2, accession no. K03455) and HIV-2 (accession no. KX174313) were used for subtyping and as outgroup, respectively. Phylogenetic tree was constructed by using the neighbor-joining method (MEGA6 program), and the bootstrapping method was used to perform statistical evaluations.

### Statistical analysis

The Mann-Whitney U test was used to analyze continuous variables. The chi-squared test was performed for 3 × 2 and 6 × 2 contingency tables, and the Fisher’s exact test was performed for 2 × 2 contingency tables. Spearman’s rank correlation coefficient was calculated for 2 × 2 contingency tables. All analyses were performed using JMP software version 11.2.1 (SAS Institute Inc., Cary, NC). All statistical analyses were conducted with a significance level of α = 0.05 (*P* < 0.05).

## Results

### Clinical characteristics

Clinical characteristics of the patients are shown in Table 1. The median age was 42 years, and 98% of the patients (*n* = 196) were men. Twenty-seven percent of the patients (*n* = 53) were over the age of 50. The majority of the patients infected with HIV-1 (99%, *n* = 198) were Japanese; the remaining two patients were from Latin America and Oceania, respectively. Six percent of the patients (*n* = 12) were naïve to ART.

**Table 1:** Characteristics of participants at entry

Age (year), median [IQR]	42	[35–51]
Males (*n*, %)	196	(98%)
Assumed route of HIV-1 infection (*n*, %)		
Homosexual	168	(84%)
Heterosexual	28	(14%)
Others	4	(2%)
Nationality (*n*, %)		
Japanese	198	(99%)
Non-Japanese	2	(1%)
Current ART use (*n*, %)		
Naïve	12	(6%)
Use for ≥1 years	188	(94%)

Abbreviations: *HIV-1* = human immunodeficiency virus 1, *ART* = antiretroviral therapy, *IQR* = interquartile range

### Plasma IFN-γ and IL-6 measurements

**Fig. 1:**
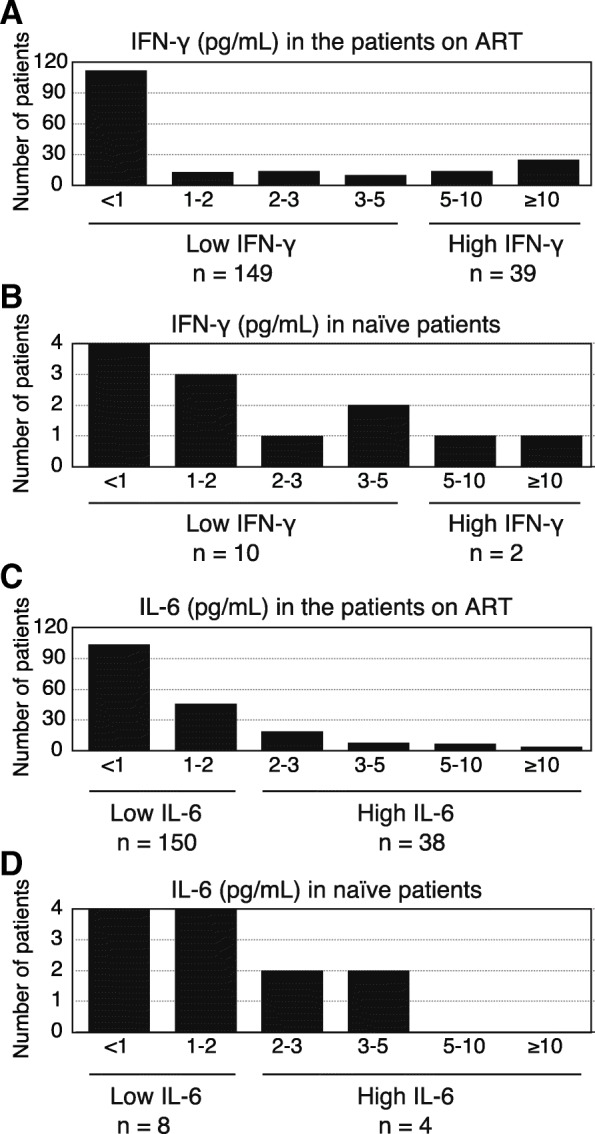
Histogram plots of the numbers of patients with different plasma cytokine levels. (**a**) IFN-γ levels in the patients on ART. (**b**) Plasma IFN-γ levels in naïve patients. (**c**) Plasma IL-6 levels in the patients on ART. (**d**) Plasma IL-6 levels in naïve patients

Plasma IFN-γ and IL-6 levels measured by ELISA are shown in Fig. 1. High IFN-γ and IL-6 levels were observed among the patients both on ART (Fig. 1a and c) and naïve to ART (Fig. 1b and d). Based on the cutoff value of 5 pg/mL, 41 patients were categorized as high IFN-γ group (21, 95% confidence interval: 15–27%), and the remaining 159 patients were categorized as low IFN-γ group. Furthermore, 42 patients (21, 95% confidence interval: 16–27%) had plasma IL-6 ≥ 2 pg/mL; among them, 17 patients also had IFN-γ ≥ 5 pg/mL. The distributions of patients between high and low cytokine groups were different for IFN-γ and IL-6 (*P* < 0.0001).

### Reproducibility of the initial high IFN-γ measurements

Reproducibility of the test results was examined by measuring plasma concentrations of IFN-γ three times in 35 out of 41 patients in the high IFN-γ group. IFN-γ concentration was ≥5 pg/mL in the two follow-up measurements for all patients who had IFN-γ ≥ 10 pg/mL at the initial measurement (*n* = 24, 69%). Similarly, IFN-γ concentration was ≥5 pg/mL in the two follow-up measurements for 6 of the 11 patients whose IFN-γ was 5–10 pg/mL at the initial measurement. For the remaining five patients, IFN-γ concentration was 2–5 pg/mL in at least one of the two follow-up measurements. Thus, the high levels of plasma IFN-γ were consistent and not transient across the three replicate measurements in most patients (*n* = 30, 86%).

### Comparison of patient characteristics across the groups

Patients were divided into the two groups based on the initial levels of IFN-γ and IL-6, and their characteristics were compared. In order to match the characteristic background, the patients on ART were included for comparison (*n* = 188). Table 2 shows patient characteristics for each group. The median age of the patients at the time of study entry was statistically different (*P* = 0.0349), and the proportion of the patients below the age of 50 was higher in the high IFN-γ group (*P* = 0.0051). The proportion of patients who were on a protease inhibitor was significantly lower in the high IFN-γ group (26%) than in the low IFN-γ group (44%, *P* = 0.0449). The duration of ART in the high IL-6 group was shorter than that in the low IL-6 group. Patients in the high IFN-γ group were less likely to have dyslipidemia (15%, *n* = 6) compared with those in the low IFN-γ group (33%, *n* = 49; *P* = 0.0476). In addition, patients in the high IL-6 group had a higher rate of diabetes mellitus and higher levels of serum C-reactive protein than those in the low IL-6 group (*P* = 0.0266 and 0.0073, respectively). These observations indicated that patients’ characteristics were different in the groups with high and low IFN-γ and IL-6 levels.

**Table 2:** Comparison of the groups with high and low IFN-γ and IL-6 levels among the patients on ART at entry

	High IFN-γ	Low IFN-γ	*P*-value	High IL-6	Low IL-6	*P*-value
N	39	149		38	150	
Age (year), median [IQR]	40 [33–44]	43 [36–52]	0.0349*	45 [37–53]	42 [35–51]	0.2683
Age < 50 (*n*, %)	35 (90%)	101 (68%)	0.0051*	25 (66%)	111 (74%)	0.3166
Males (*n*, %)	39 (100%)	146 (98%)	1.000	38 (100%)	147 (98%)	1.000
Assumed route of HIV-1 infection (*n*, %)			0.7044			0.1995
Homosexual	34 (87%)	123 (83%)		35 (92%)	122 (81%)	
Heterosexual	4 (10%)	23 (15%)		2 (5%)	25 (17%)	
Others	1 (3%)	3 (2%)		1 (3%)	3 (2%)	
Japanese (*n*, %)	38 (97%)	148 (99%)	0.3727	38 (100%)	148 (99%)	1.0000
CD4^+^ cell count (cell/μL), median [IQR]	491 [396–659]	535 [443–736]	0.271	531 [419–711]	538[442–735]	0.5293
HIV-1-RNA level (copies/mL), median [IQR]	< 20 [< 20–< 20]	< 20 [< 20–< 20]	0.5856	< 20 [< 20–< 20]	< 20 [< 20–< 20]	0.9208
Abacavir use (*n*, %)	7 (18%)	52 (35%)	0.0523	9 (24%)	50 (33%)	0.3285
Tenofovir use (*n*, %)	30 (77%)	92 (62%)	0.0911	28 (74%)	94 (63%)	0.2548
Protease inhibitor use (*n*, %)	10 (26%)	65 (44%)	0.0449*	14 (37%)	61 (41%)	0.7140
Darunavir use (*n*, %)	10 (26%)	51 (34%)	0.3428	9 (24%)	52 (35%)	0.2458
Atazanavir use (*n*, %)	0 (0%)	4 (3%)	0.5820	1 (3%)	3 (2%)	1.0000
Lopinavir use (*n*, %)	0 (0%)	5 (4%)	0.5854	3 (8%)	2 (1%)	0.0571
Fosamprenavir use (*n*, %)	0 (0%)	5 (3%)	0.5854	1 (3%)	4 (3%)	1.0000
Integrase inhibitor use (*n*, %)	24 (62%)	73 (49%)	0.2079	24 (63%)	73 (49%)	0.1457
Duration of ART (year), median [IQR]	3.8 [1.8–6.3]	4.4 [2.4–6.6]	0.2283	3.4 [1.8–5.9]	4.4 [2.5–6.7]	0.0438*
Frequency of ART exchange, median [IQR]	0 [0–1]	1 [0–2]	0.4936	0 [0–1]	1 [0–2]	0.0607
Comorbidities						
Chronic renal disease (*n*, %)	3 (8%)	18 (12%)	0.5747	2 (5%)	19 (13%)	0.2572
Chronic hepatitis B (*n*, %)	3 (8%)	13 (9%)	1.0000	2 (5%)	14 (9%)	0.5333
Dyslipidemia (*n*, %)	6 (15%)	49 (33%)	0.0476*	12 (31%)	43 (29%)	0.6957
Statin use (*n*, %)	1 (3%)	13 (9%)	0.3072	1 (3%)	13 (9%)	0.3078
Hypertension (*n*, %)	3 (8%)	18 (12%)	0.5747	6 (16%)	15 (10%)	0.3850
Diabetes mellitus (*n*, %)	1 (3%)	12 (8%)	0.3088	6 (16%)	7 (5%)	0.0266*
Laboratory test						
Serum C-reactive protein (mg/dL)	0.05 [0.02–0.10]	0.06 [0.03–0.17]	0.2224	0.10 [0.04–0.46]	0.05 [0.03–0.11]	0.0073*

Abbreviations: *IQR* = interquartile range, *HIV-1* = human immunodeficiency virus 1, *ART* = Antiretroviral therapy

*Significant difference

The rates of comorbidities were also evaluated at the time of HIV-1 diagnosis (Table 3). The rate of dyslipidemia at diagnosis was lower than that at entry (9% versus 29%; *P* < 0.0001). Similar results regarding the rates of hypertension (2% versus 11%; *P* = 0.0006) and chronic renal disease (2% versus 11%; *P* = 0.0002, respectively) were observed at diagnosis and at entry. Dyslipidemia rates between the high and low IFN-γ groups and the rates of diabetes mellitus between the high and low IL-6 groups (*P* = 0.5327 and *P* = 0.0635, respectively) were not significantly different. These observations indicate that comorbidities tended to appear during the course of HIV infection.

**Table 3:** Comparison of the groups with high and low IFN-γ and IL-6 levels among the patients on ART at diagnosis of HIV infection

	High IFN-γ	Low IFN-γ	*P*-value	High IL-6	Low IL-6	*P*-value
Clinical categories			0.7550			0.4122
Acute HIV infection	7 (18%)	26 (17%)		4 (11%)	29 (19%)	
Chronic HIV infection, Category A/B	21 (54%)	89 (60%)		25 (66%)	85 (57%)	
Category C	11 (28%)	34 (23%)		9 (24%)	36 (24%)	
CD4^+^ cell count (cell/μL), median [IQR]	265 [71–393]	253 [116–393]	0.9986	261 [107–386]	253 [104–398]	0.6920
HIV-1-RNA level (copies/mL), median [IQR]	96,750 [25,050–789,500]	105,000 [26,725–462,250]	0.9956	79,150 [25,475–373,250]	101,500 [26,100–491,500]	0.5009
Comorbidities						
Chronic renal disease (*n*, %)	0 (0%)	3 (2%)	1.0000	1 (2%)	2 (1%)	0.5089
Chronic hepatitis B (*n*, %)	2 (5%)	12 (8%)	0.7391	2 (5%)	12 (8%)	0.7384
Dyslipidemia (*n*, %)	2 (5%)	15 (9%)	0.5327	3 (7%)	14 (9%)	1.0000
Hypertension (*n*, %)	0 (0%)	4 (3%)	0.5833	2 (5%)	2 (1%)	0.1949
Diabetes mellitus (*n*, %)	0 (0%)	5 (3%)	0.5855	3 (7%)	2 (1%)	0.0635

Abbreviations: *IQR* = interquartile range, *HIV-1* = human immunodeficiency virus 1

Next, Spearman’s rank correlation coefficients among the factors related to high IFN-γ levels (age < 50, abacavir use, protease inhibitor use, and with dyslipidemia at entry) were calculated (Table 4). In contrary to the results illustrated in Table 2, a significant association was observed between high IFN-γ and abacavir use. A significant relationship was observed in all other comparisons except those between protease inhibitor use and abacavir use as well as between protease inhibitor use and age < 50, suggesting that these factors may interact with each other.

**Table 4:** Spearman’s rank correlation coefficient indicating the presence or absence of the correlation between the factors related to high IFN-γ levels

	Age < 50	Abacavir use	Protease inhibitor use	With dyslipidemia
High IFN-γ	0.199 *P* = 0.0062*	−0.1481 *P* = 0.0425*	−0.1489 *P* = 0.0414*	− 0.1560 *P* = 0.0325*
Age < 50		− 0.2993 *P* < 0.0001*	0.0181 *P* = 0.8054	−0.2297 *P* = 0.0015*
Abacavir use			0.0108 *P* = 0.8827	0.3462 *P* < 0.0001*
Protease inhibitor use				0.1924 *P* = 0.0082*

*Significant difference

### Recovery of CD4^+^ cell counts following ART

**Fig. 2:**
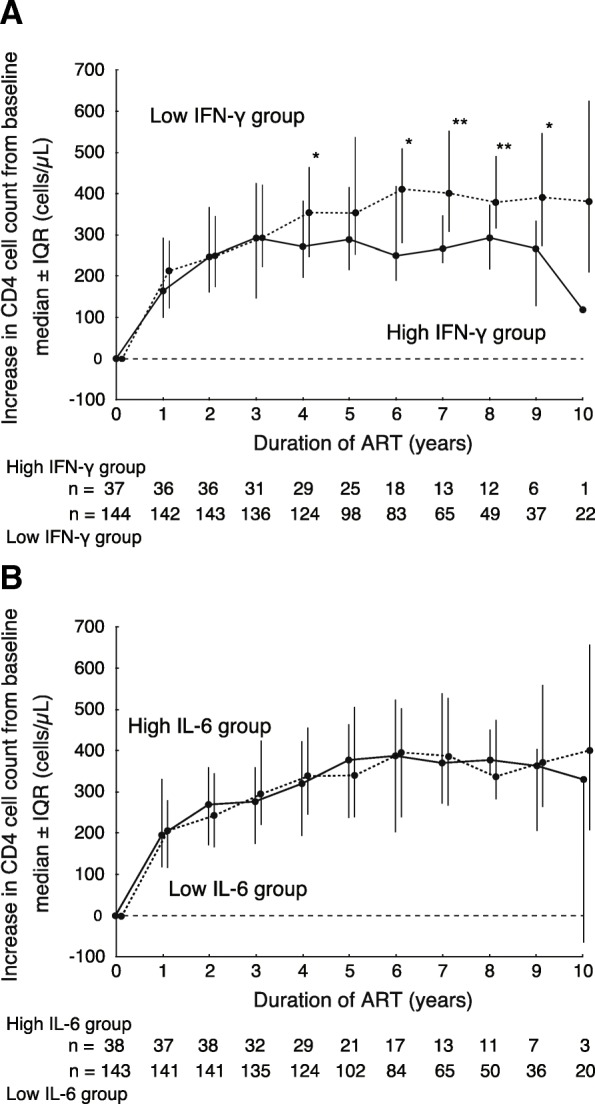
Increase in the CD4^+^ cell count from the start of ART (baseline). (**a**) The increase in CD4^+^ cell count from the baseline is expressed as the median ± interquartile range (IQR). Solid and dotted lines indicate the high and low IFN-γ groups, respectively. (**b**) The increase in CD4^+^ cell count in the high and low IL-6 groups. The total number of patients at respective time points is indicated below each graph. *P < 0.05, **P < 0.01

**Fig. 3:**
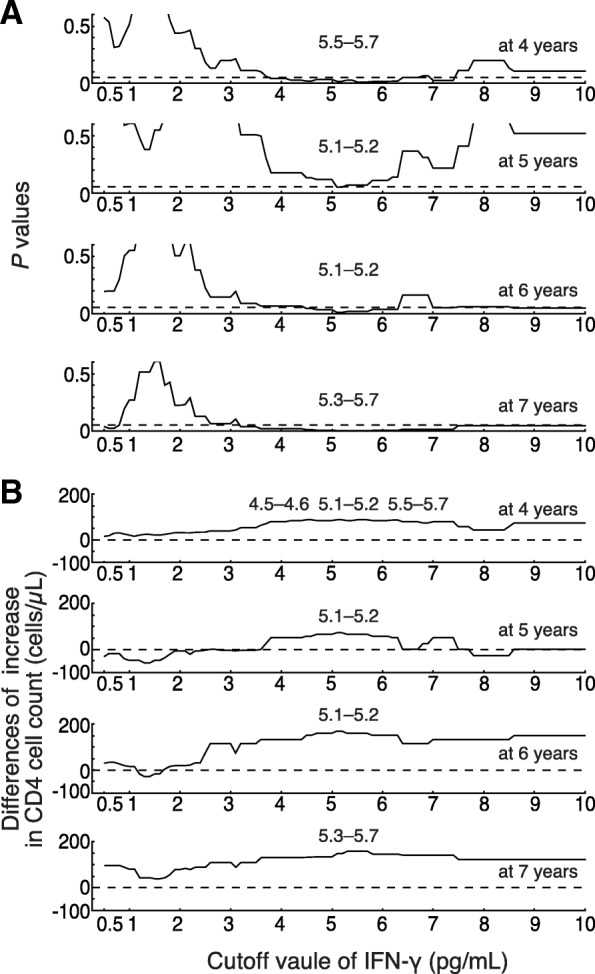
Evaluation of cutoff values for plasma IFN-γ concentration using the differences of increase in the CD4^+^ cell count from the start of ART (baseline). (**a**) Cutoff values for plasma IFN-γ concentration were increased by 0.1 from 0.5 to 10. The participants were divided into two group and group comparisons were performed. *P* values of the Mann-Whitney U test are indicated. The range of cutoff values with the smallest *P* value are shown in the top of figures. (**b**) The differences of increase in CD4^+^ cell count between two groups. The range of cutoff values with the maximum differences is shown in the top of figures

The recovery of CD4^+^ cell counts following ART was compared between the groups (Fig. 2). Patients that did not receive ART (*n* = 12) or for which CD4^+^ cell count just before initiating ART was unknown (*n* = 7) were excluded from this analysis. At the initiation of ART (baseline), CD4^+^ cell counts were not significantly different between the groups, with 268 and 210 cells/μL in the high and low IFN-γ groups (*P* = 0.0961), and 229 and 217 cell/μL in the high and low IL-6 groups (*P* = 0.9112), respectively. Figure 2 shows the median CD4^+^ cell count increase ± interquartile range (IQR) from the baseline. The difference between the groups was not significant for 3 years on ART. However, there was a significant difference indicating lower recovery of CD4^+^ cell counts in the high IFN-γ group after 4 years of ART (Fig. 2a). A similar reduction was not observed in the high IL-6 group (Fig. 2b). Cutoff values were evaluated by the comparisons with CD4^+^ cell count recovery after 4–7 years of ART. Cutoff values varied from 0.5, which was close to the median value of plasma IFN-γ concentration (0.54) in this study, to 10, so the participants were divided into two groups using these cutoff values. Figure 3 indicates *P* values and differences in the increase of CD4^+^ cell count for intergroup comparisons. The lowest *P* values were located within 5.1–5.7 pg/mL of IFN-γ concentration in all four assays (Fig. 3a). Maximum differences were also located within 4.5–5.7 pg/mL (Fig. 3b). These observations suggested that 5 pg/mL was potentially the optimal cutoff value for IFN-γ concentration and higher cutoff value may show nearly the same results. No significant differences were observed in any cutoff values at 1–3 years of ART for plasma IFN-γ concentration and at 1–10 years of ART for plasma IL-6 concentration.

### Phylogenetic analysis

**Fig. 4:**
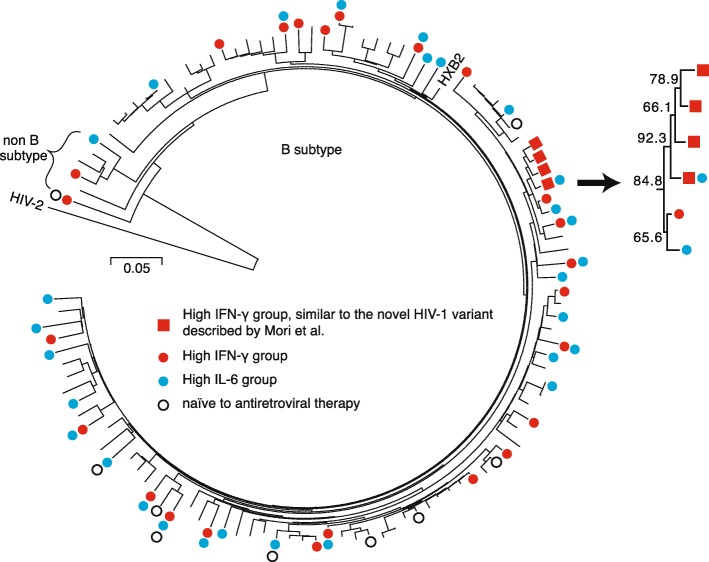
Phylogenetic analysis of HIV-1 genes encoding protease and reverse transcriptase residues. HIV-1197-bp and 720-bp long sequences of genes encoding, respectively, protease and reverse transcriptase were analyzed. Red circles and boxes indicate patients in the high IFN-γ group. Blue circles indicate patients in the high IL-6 group. Sequences from four patients, indicated by red boxes, had an insertion of the amino acid sequence Q[S/N]RPE in the p6 region of *gag* and an addition of the amino acid sequence QNME in the C′-terminus of integrase. Sequences from six patients, including these four patients, formed a cluster with a bootstrapping value of 84.8%, and the details are shown in the inset

Phylogenetic analysis of HIV-1 variants was performed in 113 patients by comparing 197-bp and 720-bp long sequences of genes encoding protease and reverse transcriptase, respectively (Fig. 4). Among the 45 patients with high levels of plasma IFN-γ and/or IL-6, 39 were distributed in a scattered manner within B subtype and non-B subtype. The sequences from the remaining six patients formed a cluster with a bootstrapping value of 84.8%. Sequences from four out of these six patients formed a cluster with a bootstrapping value of 92.3%; they exhibited similarity to the sequence previously described by Mori et al., which was a novel HIV-1 variant with rapid disease progression during primary HIV-1 infection [16]. Specifically, there was an insertion of the amino acid sequence Q[S/N]RPE in the p6 region of *gag* in addition to the amino acid sequence QNME in the C′-terminus of integrase. These observations suggested a possibility that viral factors might be involved in the maintenance of high plasma IFN-γ levels in these patients.

## Discussion

In the present study, we determined the proportion of HIV-1-infected patients with a high level of plasma IFN-γ (≥5 pg/mL) and examined their clinical characteristics. We demonstrated that the patients with a high plasma IFN-γ level were more likely to be younger, and less likely to have dyslipidemia or to be on a protease inhibitor. The incidence rate of comorbidities, such as diabetes and hypertension, in the high IFN-γ group was equivalent or nominally lower than that of the low IFN-γ group, although significant differences were not observed. Most of these results were not qualitatively similar to the findings reported in patients with high levels of circulating IL-6 in this and previous studies [10]. Borges et al. performed an analysis of 9864 patients with virological suppression and demonstrated that the high level of plasma IL-6 was associated with multiple factors that affect inflammation, such as old age, use of protease inhibitors, comorbidities, reduced renal function, and others. Some studies have also demonstrated associations between the high level of circulating IL-6 and various clinical outcomes, including death and the onset of acquired immune deficiency syndrome [9, 11, 17]. Our findings support a possible association between the high level of IFN-γ with endogenous anti-HIV response rather than with inflammation [8].

Notably, HIV-1-infected patients with a high plasma IFN-γ level had reduced CD4^+^ cell count recovery from year 4 on ART and onwards than patients with a low plasma IFN-γ level. Various factors have been shown to be associated with suboptimal CD4^+^ cell count recovery, including low nadir/baseline CD4^+^ cell count, old age, male gender, prolonged duration between HIV-1 infection and the initiation of ART, hepatitis C virus infection, hepatitis B virus infection, comorbidities, injection drug use, up-regulation of surface markers of lymphocyte activation, and gene polymorphisms of *IL-10* and *IL7RA* [18–37]. In addition, many soluble biomarkers have been also examined for the association with suboptimal recovery of CD4^+^ cell count [38, 39]. However, the nadir/baseline CD4^+^ cell count may be a confounding factor as it is strongly associated with suboptimal recovery of CD4^+^ cell count and influences the circulating level of soluble biomarkers. After controlling for that confounding factor, only a limited number of biomarkers, including IL-6 before ART and interferon-inducible protein-10 after ART, were associated with the suboptimal recovery of CD4^+^ cell count [34, 35]. In the present study, we demonstrated that there was no significant difference in CD4^+^ cell counts prior to and 3 years after ART between the low and high IFN-γ groups. Unexpectedly, the proportion of patients who were considered at high risk of reduced CD4^+^ cell count recovery, such as older patients and those with comorbidities, was lower in the high IFN-γ group than in the low IFN-γ group. Collectively, these findings suggest that a high level of plasma IFN-γ may be an independent factor associated with lower recovery of CD4^+^ cell count.

Our phylogenetic analysis demonstrated that HIV-1 genetic sequence in patients with a high IFN-γ level was distributed among different subtypes in a scattered manner, indicating that gene polymorphisms may be one of the causative factors of high plasma IFN-γ level. IFN-γ + 874 T/A gene polymorphism is located within the first intron and encompasses the binding site for the transcription factor NF-κB. Binding affinity of NF-κB for the + 874 A-allele is lower, and it results in reduced production of IFN-γ. Because it has been reported that individuals with + 874 A-allele may be at a higher risk of tuberculosis [40], many studies examined the association between this polymorphism and various diseases. In particular, it has been shown that the A-allele was a risk factor for HIV infection [41], and that HIV-infected patients with the A-allele had a higher risk of HIV-tuberculosis co-infection [42], lower CD4^+^ cell count [31, 43], and lack of response to ART [44]. Our findings were not in accordance with previous reports, which demonstrated that patients with the A-allele, associated with lower IFN-γ production, showed a reduction in CD4^+^ cell count. These findings suggest that IFN-γ gene polymorphism may not be the primary factor that contributes to the high level of circulating IFN-γ.

In the phylogenetic analysis, we identified four patients that formed a cluster (red boxes in Fig. 4). In addition to the similarities of their genetic sequence, they had common geographical and chronological features, as all four of them were diagnosed in Osaka after 2011. Thus, these patients were likely to have been infected by the novel HIV-1 variant previously described by Mori et al. [16]. It is still unclear why the patients who were infected with this novel variant had accelerated disease progression. Further investigations of this phenomenon are required because there may be a potential common mechanism underlying the accelerated disease progression and high plasma IFN-γ level.

There were several limitations in our study: it included a relatively small number of patients, especially of those infected with novel HIV-1 variant previously described [16]. Furthermore, information on some factors that might affect circulating cytokines, such as consumption of tobacco and alcohol [10], were missing. In addition, the data on CD4^+^ cell counts were collected retrospectively, and plasma IFN-γ level was evaluated in a cross-sectional manner. Although we selected the cutoff value using earlier data [7], evaluation of the cutoff value in this study using the differences of the recovery of CD4^+^ cell count after 4 years of ART suggested that 5 pg/mL of IFN-γ concentration was a potential candidate for optimal cutoff value. In addition, our study did not investigate IFN-γ-producing cells. Further studies are required to uncover the biological mechanism contributing to the high level of circulating IFN-γ.

## Conclusions

Twenty-one percent of HIV-1-infected patients, who were either naïve to ART (CD4^+^ cell count > 200 cells/μL) or had achieved viral suppression after ART for 1 year or longer, were found to have a high (> 5 pg/mL) level of plasma IFN-γ. These patients did not have a higher rate of comorbidities associated with immune activation during the course of HIV infection. However, they demonstrated reduced CD4^+^ cell count recovery after 4 years on ART, indicating that these patients may be influenced by persistent immune activation.

## Additional file

Additional file 1: **S1 File.** Supplementary Methods. (DOCX 32 kb)

## Abbreviations

ART: antiretroviral therapy
ELISA: enzyme-linked immunosorbent assay
IFN-γ: interferon-γ
IL-6: interleukin-6
IQR: interquartile range.
