# Supplementary material for: Clinical characteristics of HIV-1-infected patients with high levels of plasma interferon-γ: a multicenter observational study
Source: BMC Infect Dis. 2019 Jan 5;19:11. doi: 10.1186/s12879-018-3643-2 (PMC6321664; doi:10.1186/s12879-018-3643-2)
Supplement: Supplementary file 1 — S1 File. Supplementary Methods. (DOCX 32 kb) [file 12879_2018_3643_MOESM1_ESM.docx]

**Supplementary Materials and Methods**

***Nucleotide sequence accession number***

Sequences used in this study are available in GenBank under the following accession numbers:

AB866767, AB866768, AB866778, AB866780, AB866782, AB866796, AB866810, AB866811, AB866816, AB866835, AB866841, AB866873, AB866890, AB866922, AB866932, AB866940, AB866942, AB866951, AB866957, AB866974, AB866980, AB867002, AB867003, AB867027, AB867030, AB867039, AB867042, AB867045, AB867074, AB867112, AB867120, AB867133, AB867143, AB867144, AB867147, AB867153, AB867162, AB867168, AB867175, AB867180, AB867181, LC163331, LC163351, LC163353, LC163371, LC163381, LC163386, LC163389, LC163395, LC163415, LC163421, LC163426, LC163428, LC163437, and LC371690–371748.
